# Supplementary figures and images for: Analysis-ready VCF at Biobank scale using Zarr
Source: Gigascience. 2025 Jun 2;14:giaf049. doi: 10.1093/gigascience/giaf049 (PMC12127038; doi:10.1093/gigascience/giaf049)

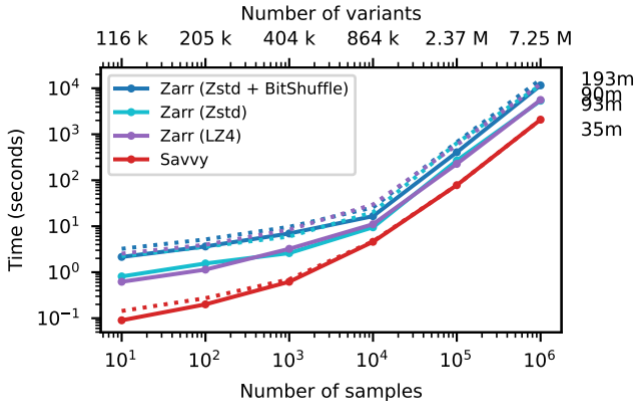

Supplement: giaf049_Supplemental_Files [file giaf049_supplemental_files.zip › FigS1.pdf]

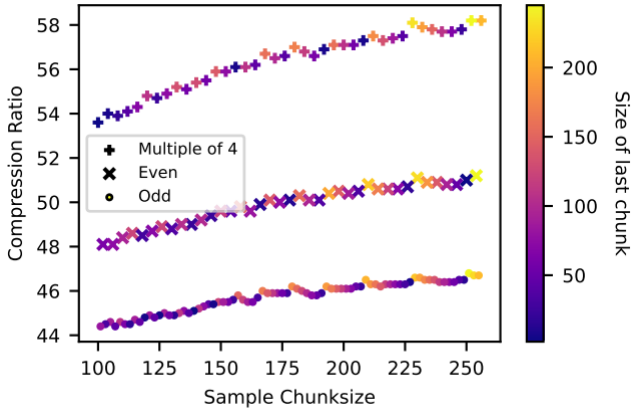

Supplement: giaf049_Supplemental_Files [file giaf049_supplemental_files.zip › FigS10.pdf]

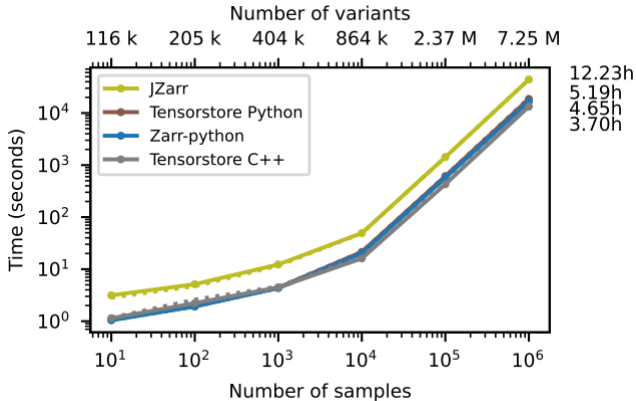

Supplement: giaf049_Supplemental_Files [file giaf049_supplemental_files.zip › FigS2.pdf]

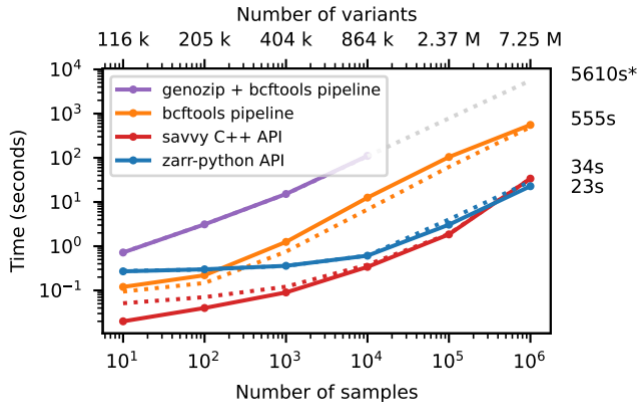

Supplement: giaf049_Supplemental_Files [file giaf049_supplemental_files.zip › FigS3.pdf]

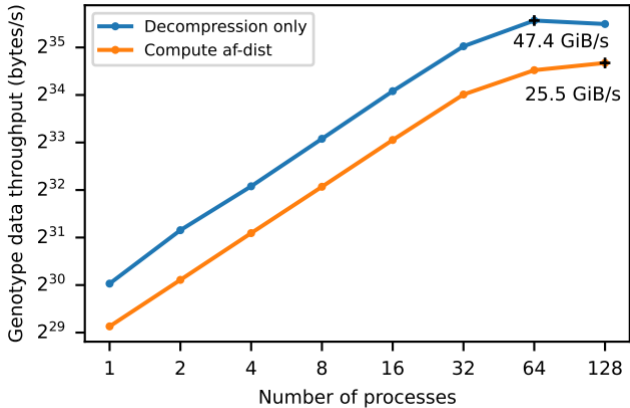

Supplement: giaf049_Supplemental_Files [file giaf049_supplemental_files.zip › FigS4.pdf]

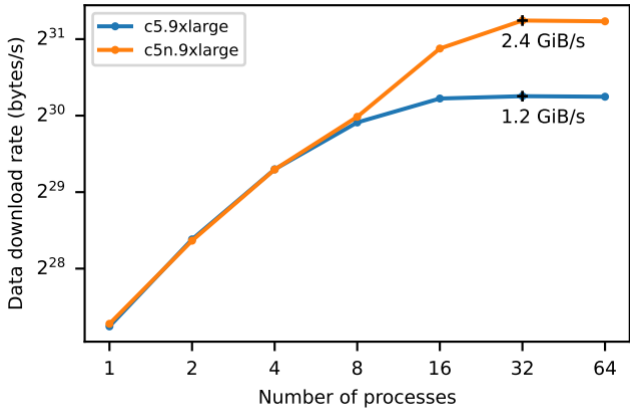

Supplement: giaf049_Supplemental_Files [file giaf049_supplemental_files.zip › FigS5.pdf]

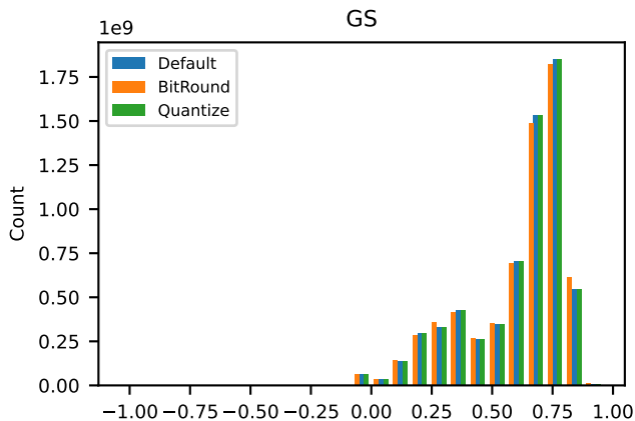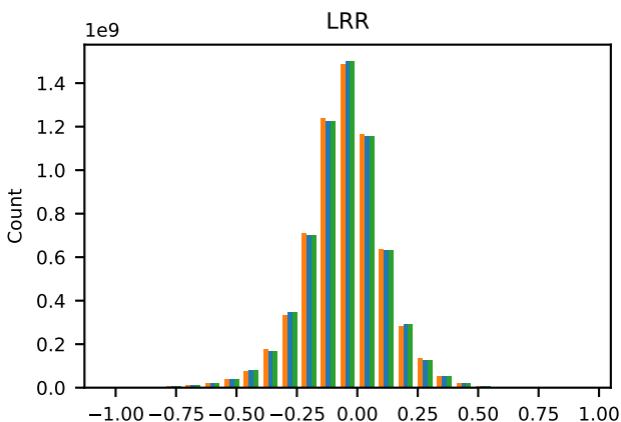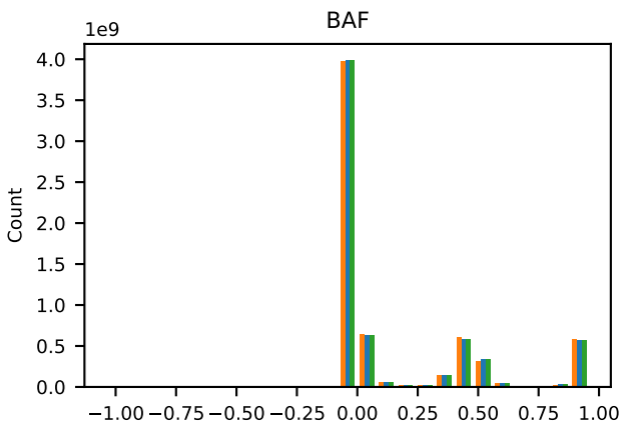

Supplement: giaf049_Supplemental_Files [file giaf049_supplemental_files.zip › FigS6.pdf]

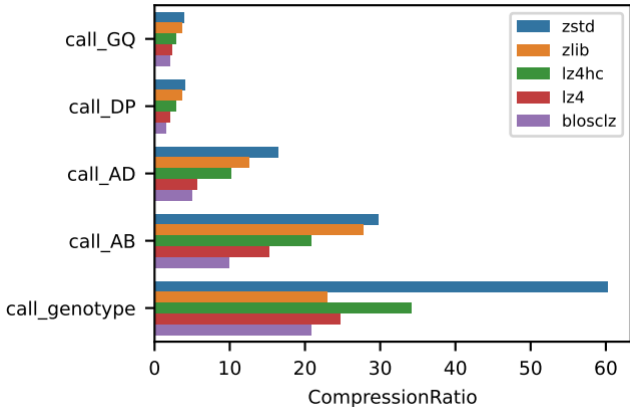

Supplement: giaf049_Supplemental_Files [file giaf049_supplemental_files.zip › FigS7.pdf]

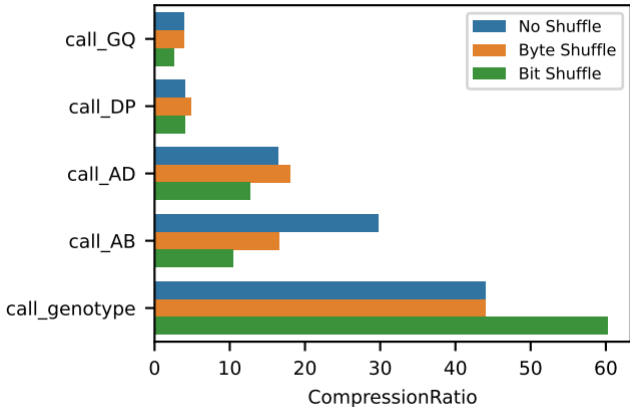

Supplement: giaf049_Supplemental_Files [file giaf049_supplemental_files.zip › FigS8.pdf]

(A)

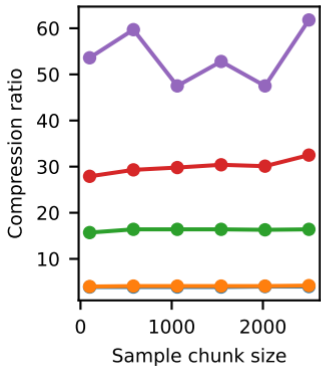

(B)

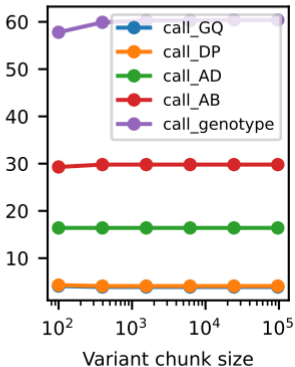

Supplement: giaf049_Supplemental_Files [file giaf049_supplemental_files.zip › FigS9.pdf]
