# Supplementary material for: Analysis-ready VCF at Biobank scale using Zarr
Source: Gigascience. 2025 Jun 2;14:giaf049. doi: 10.1093/gigascience/giaf049 (PMC12127038; doi:10.1093/gigascience/giaf049)
Supplement: giaf049_Reviewer_1_Report_original_submission — Nezar Abdennur -- 8/7/2024 [file giaf049_reviewer_1_report_original_submission.pdf]

Reviewer Report

Title: Analysis-ready VCF at Biobank scale using Zarr

Version: Original Submission **Date:** 8/7/2024

Reviewer name: Nezar Abdennur

Reviewer Comments to Author:

The authors present VCF Zarr, a specification that translates the variant call format (VCF) data model into an array-based representation for the Zarr storage format. They also present the `vcf2zarr` utility to convert large VCFs to Zarr. They provide data compression and analysis benchmarks comparing VCF Zarr to existing variant storage technologies using simulated genotype data. They also present a case study on real world Genomics England aggV2 data.The authors' benchmarks overall show that VCF Zarr has superior compression and computational analysis performance at scale relative to data stored as row-oriented VCF and that VCF Zarr is competitive with specialized storage solutions that require similarly specialized tools and access libraries for querying. An attractive feature is that VCF Zarr allows for variant annotation workflows that do not require full dataset copy and conversion. Another key point is that Zarr is a high-level spec and data model for the chunked storage of n-d arrays, rather than a byte-level encoding designed specifically around the genomic variant data type. I personally have used Zarr productively for several applications unrelated to statistical genetics. While Zarr VCF mildly underperforms some of the specialized formats (Savvy in compute, Genozip in compression) in a few instances, I believe the accessibility, interoperability, and reusability gains of Zarr make the small tradeoff well worthwhile.Because Zarr has seen heavy adoption in other scientific communities like the geospatial and Earth sciences, and is well integrated in the scientific Python stack, I think it holds potential for greater reusability across the ecosystem. As such, I think the VCF Zarr spec is a highly valuable if not overdue contribution to an entrenched field that has recently been confronted by a scalability wall.Overall, the paper is clear, comprehensive, and well written. Some high-level comments:* The benefits for large scientific datasets to be analysis-ready cloud-optimized (ARCO) have been well articulated by Abernathey et al., 2021. However, I do think that the "local"/HPC single-file use case is still important and won't disappear any time soon, and for some file system use cases, expansive and deep hierarchies can be performance limiting (this was hinted at in one of the benchmarks). In this scenario would a large Zarr VCF perform reasonably well (or even better on some file systems) via a single local zip store?* The description of the intermediate columnar format (ICF) used by `vcf2zarr` is missing some detail. At first I got the impression it might be based on something like Parquet, but running the provided code showed that it consists of a similar file-based chunk layout to Zarr. This should be clarified in the manuscript.* The authors discuss the possibility of storing an index mapping genomic coordinates to chunk indexes. Have Zarr-based formats in other fields like geospatial introduced their own indexing approaches to take inspiration from?* Since VCF Zarr is still a draft proposal, it could be useful to indicate where community discussions are happening and how potential new contributors can get involved, if possible. This doesn't need to be in the paper per se, but perhaps documented in the spec repo.Minor comments:* In the background: "For the representation to be FAIR,

it must also be accessible," -- A is for "accessible", so "also" doesn't make sense.* "There is currently no efficient, FAIR representation...". Just a nit and feel free to ignore, but the solution you present is technically "current".* In Figure 2, the zarr line is occluded by the sav line and hard to see.

Level of Interest

Please indicate how interesting you found the manuscript: Choose an item.

Quality of Written English

Please indicate the quality of language in the manuscript: Choose an item.

Declaration of Competing Interests

Please complete a declaration of competing interests, considering the following questions:

- Have you in the past five years received reimbursements, fees, funding, or salary from an organisation that may in any way gain or lose financially from the publication of this manuscript, either now or in the future?
- Do you hold any stocks or shares in an organisation that may in any way gain or lose financially from the publication of this manuscript, either now or in the future?
- Do you hold or are you currently applying for any patents relating to the content of the manuscript?
- Have you received reimbursements, fees, funding, or salary from an organization that holds or has applied for patents relating to the content of the manuscript?
- Do you have any other financial competing interests?
- Do you have any non-financial competing interests in relation to this paper?

If you can answer no to all of the above, write 'I declare that I have no competing interests' below. If your reply is yes to any, please give details below.

I declare that I have no competing interests.

I agree to the open peer review policy of the journal. I understand that my name will be included on my report to the authors and, if the manuscript is accepted for publication, my named report including any attachments I upload will be posted on the website along with the authors' responses. I agree for my report to be made available under an Open Access Creative Commons CC-BY license (http://creativecommons.org/licenses/by/4.0/). I understand that any comments which I do not wish to be included in my named report can be included as confidential comments to the editors, which will not be published.

Choose an item.

To further support our reviewers, we have joined with Publons, where you can gain additional credit to further highlight your hard work (see: https://publons.com/journal/530/gigascience). On publication of this paper, your review will be automatically added to Publons, you can then choose whether or not to claim your Publons credit. I understand this statement.

Yes Choose an item.
